# Multidirectional *in vitro* and *in cellulo* studies as a tool for identification of multi-target-directed ligands aiming at symptoms and causes of Alzheimer’s disease

**DOI:** 10.1080/14756366.2020.1835882

**Published:** 2020-10-22

**Authors:** Natalia Szałaj, Justyna Godyń, Jakub Jończyk, Anna Pasieka, Dawid Panek, Tomasz Wichur, Krzysztof Więckowski, Paula Zaręba, Marek Bajda, Anja Pislar, Barbara Malawska, Raimon Sabate, Anna Więckowska

**Affiliations:** a Department of Physicochemical Drug Analysis, Faculty of Pharmacy, Jagiellonian University Medical College, Kraków, Poland; b Department of Organic Chemistry, Faculty of Pharmacy, Jagiellonian University Medical College, Kraków, Poland; c Faculty of Pharmacy, University of Ljubljana, Ljubljana, Slovenia; d Department of Pharmacy and Pharmaceutical Technology and Physical Chemistry, Faculty of Pharmacy and Food Science, University of Barcelona, Barcelona, Spain; e Institute of Nanoscience and Nanotechnology (IN2UB), University of Barcelona, Barcelona, Spain

**Keywords:** Multifunctional ligands, β-secretase, 5-HT6 receptor antagonists, cholinesterase inhibitors

## Abstract

Effective therapy of Alzheimer’s disease (AD) requires treatment with a combination of drugs that modulate various pathomechanisms contributing to the disease. In our research, we have focused on the development of multi-target-directed ligands – 5-HT_6_ receptor antagonists and cholinesterase inhibitors – with disease-modifying properties. We have performed extended *in vitro* (FRET assay) and *in cellulo* (*Escherichia coli* model of protein aggregation) studies on their β-secretase, tau, and amyloid β aggregation inhibitory activity. Within these multifunctional ligands, we have identified compound **17** with inhibitory potency against tau and amyloid β aggregation in *in cellulo* assay of 59% and 56% at 10 µM, respectively, *h*BACE IC_50_=4 µM, *h*5TH6 *K*_i_=94 nM, *h*AChE IC_50_=26 nM, and *eq*BuChE IC_50_=5 nM. This study led to the development of multifunctional ligands with a broad range of biological activities crucial not only for the symptomatic but also for the disease-modifying treatment of AD.

## 1. Introduction

Alzheimer’s disease (AD) is among the top ten leading causes of death globally according to the World Health Organisation and among them, the only disease we cannot prevent, cure or even slow down^1^. The available therapy – targeting cholinergic (donepezil, galantamine, and rivastigmine) and glutamatergic neurotransmission (memantine) – provide only a modest efficacy on cognitive impairment^2^. Therefore, the interest in the development of new drugs, preferentially targeting processes underlying the disease, is among urgent research goals.

AD belongs to a large group of “conformational diseases,” that are caused by misfolding, aggregation, and accumulation of certain proteins in tissues, leading to their dysfunction^3^. In AD, constitute proteins – amyloid β (Aβ) and tau protein – undergo changes resulting in the formation of amyloid plaques and neurofibrillary tangles (NFTs) that accumulate outside and inside neurons, respectively. The sequence of events leading to the development of the disease is still under discussion, but there is no doubt that both these proteins are necessary for the disease to develop^4^. The aim of the effective treatment directed at these proteins is to remove plaques and tangles or to stop them from developing^5^^,^^6^.

The main strategies for targeting Aβ pathology are dedicated to decrease its production by inhibition of β-secretase (BACE1), or modulation/inhibition of γ-secretase. Also, general strategies like passive and active immunisation or non-specific aggregation inhibition have been applied^7^. The return of monoclonal antibody aducanumab to Phase IIIb safety study for the FDA approval process has recently aroused great hopes and excitement^8^^,^^9^. It works to clear the amyloid plaques in the brain and even though the experts do not consider it as a final treatment for AD, it would be a milestone in the search for the combination therapy with anti-tau compounds. Recently, it was proven that peptide-based inhibitors of Aβ aggregation reduce aggregation and self-seeding of tau suggesting that these proteins share a common epitope thus, it is worth to control aggregation of both to stop the disease^10^. It was also clarified in *in vivo* studies that the pathological role of interactions between Aβ and tau promotes neuronal dysfunction^11^. Thus, it seems reasonable to develop both Aβ and tau-targeted therapeutics.

Lack of effective therapy and the complexity of AD led to a change in the approach in the search for new anti-AD treatment. Extensive research was directed to the search for multifunctional ligands that led to the development of a large variety of compound classes targeting processes connected with causes and symptoms of AD. The identification of the most suited combination of drug targets is challenging and extremely important and should be consistent with the balanced activities against the targets of interest^12^^,^^13^. Among the anti-AD compounds in clinical trials up to 2019, the first three places belong to compounds targeting Aβ peptide, neurotransmitters system and tau protein^14^. Thus, a combination of these activities in one molecule may be a reasonable step towards effective slowing down of AD progression. The most explored multifunctional ligands combine anticholinesterase activity with anti-aggregation properties resulting from inhibition of β-secretase or modulation of γ-secretase and direct inhibition of processes of aggregation of Aβ and tau proteins^15–18^. Also, many G-protein coupled receptors are explored in combination with anticholinesterase activity, among them cannabinoid receptors CB_1_ and CB_2_^19–22^, histamine H_3_ receptors^23–25^or serotonin 5-HT_1A_^26^, 5-HT_4_, and 5-HT_6_ receptors^27–31^.

**Figure 1.**
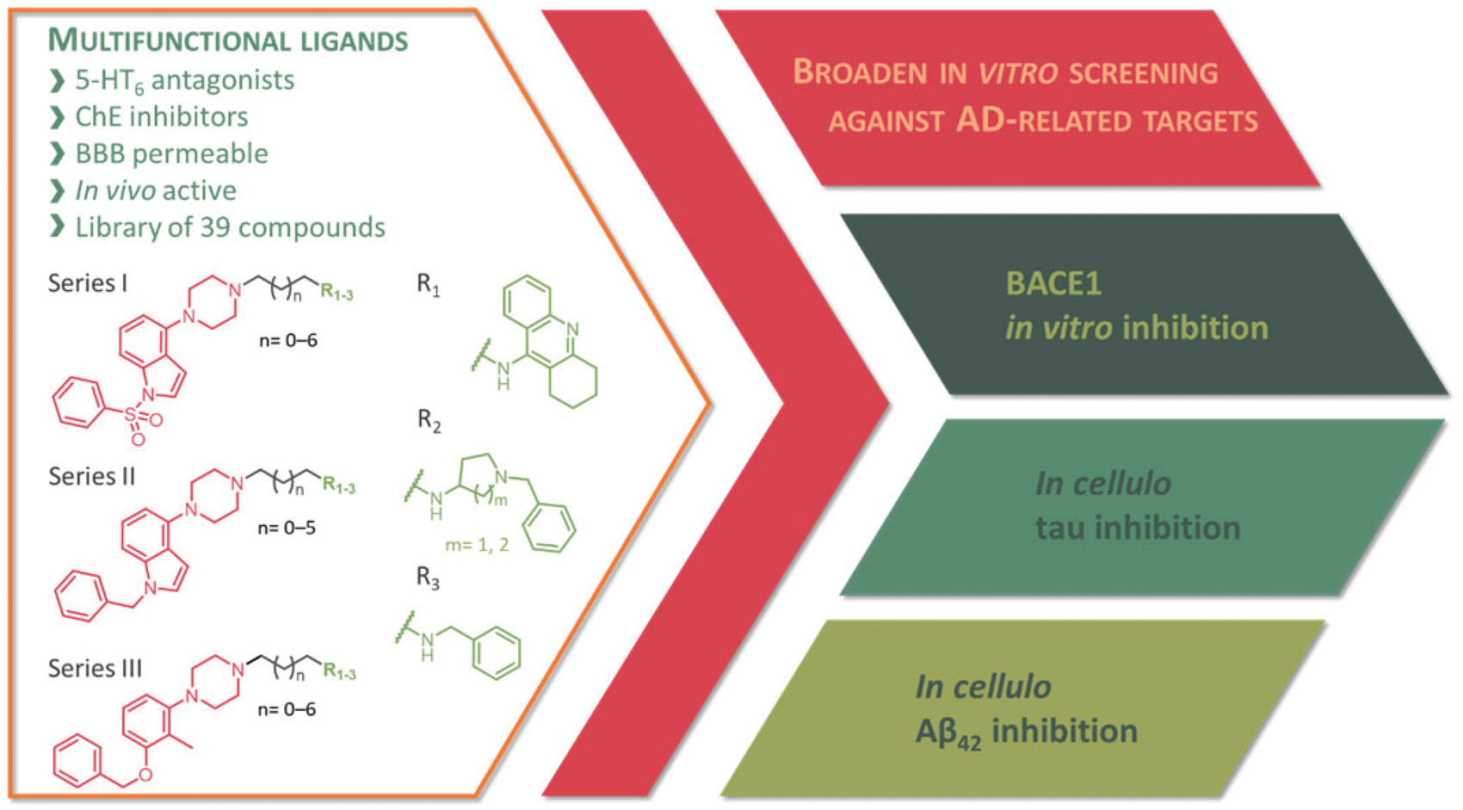
Broaden screening against Aβ and tau-related targets of a library of multifunctional 5-HT_6_ antagonists and cholinesterase inhibitors.

Here, we present the broadened *in vitro* and *in cellulo* studies on the activity of the previously published first-in-class multi-target-directed ligands (Figure 1) targeting serotonergic 5-HT_6_ receptors and cholinesterases^30^^,^^31^. Based on the results from preclinical studies, modulation of these biological targets results in an increase of acetylcholine, glutamate, noradrenaline, and dopamine in the frontal cortex and enhanced performance in a variety of paradigms of cognitive impairment in animals^32^. Moreover, clinical trials showed the superiority of combination therapy with a cholinesterase inhibitor (donepezil) and 5-HT_6_ receptor antagonists (idalopirdine) over donepezil alone^33^. Guided by this evidence, we have designed multifunctional ligands − 5-HT_6_ antagonists and cholinesterase inhibitors – that displayed well-balanced potencies against the biological targets and proved their potential in *in vivo* studies.

Since targeting aggregation processes may affect the processes underlying AD, we performed preliminary studies verifying the potential of these multifunctional ligands as inhibitors of Aβ and tau aggregation. Several of these compounds have already been tested for their anti-aggregating ability towards Aβ_42_ *in vitro*^31^ using Thioflavin T (ThT) fluorescence assay^34^. Their activity ranged from 48% to 95% at 10 µM screening concentration and, therefore, we decided to further explore their multifunctional profile and extend research on our in-house library of 39 multifunctional compounds. We have focussed on processes of aggregation by testing the compounds *in cellulo* in *Escherichia coli* cells overexpressing proteins of interest. We have verified *in vitro* results of Aβ_42_ anti-aggregating activity and tested the compounds against tau aggregation. Additionally, we have evaluated the inhibitory potential of the compounds against BACE1 – one of the key biological targets for Aβ pathology.

## 2. Results and discussion

### 2.1. Chemical profile of the compound library

The tested library of compounds (Figure 1) comprises three series of multifunctional ligands that contain pharmacophore fragments known for 5-HT_6_ receptors antagonism: 1-(phenylsulfonyl)-4-(piperazin-1-yl)-1*H*-indole (series I, Table 1), 1-benzyl-4-(piperazin-1-yl)-1*H*-indole (series II, Table 2), and 1–(3-(benzyloxy)-2-methylphenyl)piperazine (series III, Table 3). These pharmacophore fragments were combined with fragments known for their effective inhibition of cholinesterases: tacrine, *N*-benzylpiperidine (derived from donepezil), and *N*-benzylpiperidine analogues: *N*-benzylamine and *N*-benzylpyrrolidine. Among the obtained multifunctional ligands, we have selected compounds excellent for further development. They displayed not only well-balanced activities against the targets of interest but also favourable preliminary ADMET and physicochemical properties. Therefore, we decided to pursue the research and to expand the panel of tests with *in cellulo* anti-aggregating activity and *in vitro* BACE1 inhibitory activity.

**Table 1.** Inhibition of tau and Aβ_42_ aggregation and BACE1 by compounds **1**–**16** (series I).
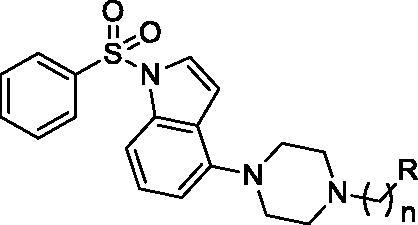

Cmpd.	*n*	R	***In cellulo*** tau inhibition %^a^	***In cellulo*** Aβ_42_ inhibition %^a^	*h*BACE1 %^b^ or IC_50_^c^ (μM)
**1**	2		58.1 ± 4.8	40.7 ± 4.3	1.58 ± 0.15
**2**	5		nd^d^	nd^d^	8.07 ± 0.15
**3**	6		37.6 ± 2.4	29.7 ± 4.0	7.31 ± 0.24
**4**	7		46.4 ± 5.9	58.8 ± 4.2	1.31 ± 0.06
**5**	8		52.7 ± 5.7	61.8 ± 4.1	2.29 ± 0.13
**6**	2		54.6 ± 6.2	64.8 ± 3.4	67.4% ± 4.8
**7**	3		39.7 ± 3.8	48.3 ± 4.2	5.82 ± 0.34
**8**	4		45.1 ± 5.1	54.5 ± 3.8	19.66 ± 1.54
**9**	5		44.8 ± 5.6	48.0 ± 4.0	23.38 ± 1.60
**10**	2		43.8 ± 5.6	18.1 ± 2.7	19.49 ± 1.18
**11**	3		32.5 ± 5.2	16.3 ± 3.3	54.2% ± 8.1
**12**	5		39.2 ± 4.5	41.4 ± 1.3	50.6% ± 10.0
**13**	2		52.7 ± 4.2	24.3 ± 3.0	6.73 ± 0.47
**14**	2		34.9 ± 5.6	32.0 ± 3.7	8.31 ± 0.51
**15**	3		58.9 ± 3.0	39.3 ± 3.7	60.2% ± 2.9
**16**	4		62.9 ± 3.5	57.2 ± 4.9	56.4% ± 6.3

^a^ The percent of inhibition at 10 µM (mean of three experiments ± SEM).

^b^ The percent of inhibition at 10 µM (mean of three experiments ± SD).

^c^ Mean of three experiments ± SEM.

^d^ Not determined.

**Table 2.** Inhibition of tau and Aβ_42_ aggregation and BACE1 by compounds **17**–**26** (series II).
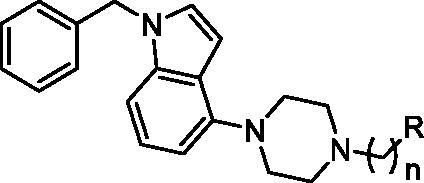

Cmpd.	*n*	*R*	*In cellulo* tau inhibition %^a^	*In cellulo* Aβ_42_ inhibition %^a^	*h*BACE1 %^b^ or IC_50_^c^ (μM)
**17**	2		58.6 ± 4.5	55.7 ± 2.8	4.09 ± 0.11
**18**	3		nd^d^	nd^d^	4.58 ± 0.21
**19**	5		nd^d^	nd^d^	7.23 ± 0.38
**20**	6		64.0 ± 5.1	66.9 ± 3.9	62.1% ± 5.8
**21**	4		41.3 ± 6.3	12.2 ± 4.1	21.88 ± 1.16
**22**	5		44.3 ± 6.5	25.2 ± 3.4	63.9% ± 7.7
**23**	6		28.4 ± 3.8	16.4 ± 2.8	30.88 ± 1.58
**24**	7		55.6 ± 4.2	21.4 ± 3.4	21.73 ± 1.57
**25**	2		27.8 ± 5.3	34.6 ± 3.0	65.7% ± 5.5
**26**	3		21.2 ± 4.9	29.9 ± 3.4	37.53 ± 1.31

^a^The percent of inhibition at 10 µM (mean of three experiments ± SEM).

^b^The percent of inhibition at 10 µM (mean of three experiments ± SD).

^c^Mean of three experiments ± SEM.

^d^Not determined.

**Table 3.** Inhibition of tau and Aβ_42_ aggregation and BACE1 by compounds **27**–**39** (series III).
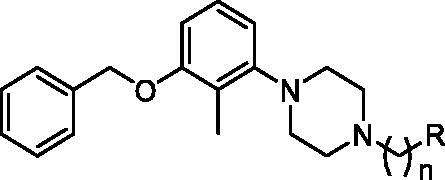

Cmpd.	*n*	*R*	*In cellulo* tau inhibition %^a^	*In cellulo* Aβ_42_ inhibition %^a^	*h*BACE1 %^b^ or IC_50_^c^ (μM)
**27**	3		64.5 ± 4.1	64.9 ± 4.5	5.76 ± 0.28
**28**	6		64.8 ± 2.2	64.2 ± 3.4	67.4% ± 11.3
**29**	7		70.3 ± 3.9	67.4 ± 4.1	4.07 ± 0.27
**30**	8		60.2 ± 2.6	66.7 ± 5.1	33.6% ± 11.1
**31**	3		39.5 ± 8.3	24.4 ± 3.4	34.0% ± 11.4
**32**	4		42.5 ± 6.3	43.2 ± 5.4	42.8% ± 12.7
**33**	5		55.8 ± 5.3	29.0 ± 4.5	31.5% ± 1.1
**34**	3		48.1 ± 7.0	25.5 ± 2.9	34.9% ± 1.8
**35**	4		45.1 ± 7.6	27.3 ± 3.1	29.6% ± 11.7
**36**	5		37.3 ± 2.7	30.4 ± 3.9	29.0% ± 6.3
**37**	2		57.3 ± 2.4	54.1 ± 4.6	40.9% ± 5.8
**38**	3		60.2 ± 3.0	55.7 ± 4.6	32.5% ± 7.8
**39**	4		62.8 ± 3.5	62.0 ± 4.0	47.0% ± 6.1
**DP-128**			68.7 ± 0.5^d^	77.5 ± 0.9^d^	–
**Inh. IV**			–	–	0.08 ± 0.01

^a^ The percent of inhibition at 10 µM (mean of three experiments ± SEM).

^b^ The percent of inhibition at 10 µM (mean of three experiments ± SD).

^c^ Mean of three experiments ± SEM.

^d^ Ref. di Pietro et al.^38^.

### 2.2. Anti-aggregating activity

We screened multifunctional ligands from our in-house library for their anti-aggregating activity against Aβ and tau in the whole bacterial cells using the Thioflavin S (ThS) fluorescence assay^35^. Bacterial cells overexpressing different proteins provide simple models that can be easily employed to monitor the mechanisms of protein aggregation related to a variety of conformational diseases^36^^,^^37^. In our assay, *E. coli* system has been implemented to study the effect of potential Aβ_42_ and tau aggregation inhibitors within intact cells. The advantage of the test over *in vitro* tests is the fact that it considers the complexity of the cellular environment that plays a crucial role in tuning protein aggregation. This assay has been validated and successfully applied to assess the anti-aggregating properties of diverse chemical structures^37^. Previously investigated tetrahydrobenzo[h][1,6] naphthyridine-6-chlorotacrine hybrid **DP-128** was used as a reference compound^38^^,^^39^.

At the 10 µM screening concentration all the compounds inhibited Aβ and tau aggregation with the following percentages of inhibition: 21–70% for Aβ and 12–67% for tau (Tables 1–3, series I–III). Among them, we identified 12 inhibitors of tau and Aβ aggregation with the inhibitory potency exceeding 50%. Most compounds with such dual inhibitory activity are tacrine (**5, 17, 20, 27**–**30**) and *N*-benzylpiperidine (**16, 37**–**39**) derivatives. Tacrine is a widely used scaffold in the AD therapy research^40^. It provides compounds with reliable inhibitory activity against cholinesterases, its derivatives are repeatedly reported as Aβ aggregation inhibitors^18^^,^^22^^,^^41^^,^^42^ and dual inhibitors of self-induced Aβ and tau aggregation potent in *in vitro* assays and living cells^43^. On the downside of the tacrine-based multifunctional ligands is the fact that very often they lack drug-likeness, mostly due to their high molecular weight, exceeded of limits logP values or potential toxicity. In this context, *N*-benzylpiperidine derived compounds, with better physicochemical properties, might be advantageous for further optimisation.

### 2.3. BACE1 inhibitory activity

To further explore the multifunctional profile of the ligands, we screened them for their inhibitory activity against human BACE1 using spectrofluorometric assay (fluorescence resonance energy transfer (FRET)-based). Past failures of BACE1 inhibitors in clinical trials may result from testing them at advanced stages of the disease. Therefore, BACE1 inhibitors are now being tested in earlier stages of AD and in asymptomatic subjects at risk of AD, with the hope of blocking the disease process before it becomes irreversible^44^.

We tested the compounds at the screening concentration of 50 µM and determined their inhibitory potency as the percentage of inhibition, which was ranging from 31% to 100% (Tables 1–3). In the next step, we determined the IC_50_ values for the compounds that inhibited enzyme at the screening concentration in more than 70%. As a reference in this assay, we have used inhibitor IV (*N*^1^-[(1*S*,2*R*)-3-(cyclopropylamino)-2-hydroxy-1-(phenylmethyl)propyl]-5-[methyl(methylsulfonyl)amino]-*N*^3^-[(1*R*)-1-phenylethyl]-1,3-benzenedicarboxamide), which is a commercially available, cell-permeable, and potent inhibitor of BACE1^45^^,^^46^. We identified 13 BACE1 inhibitors with IC_50_ values ranging from 1.31 to 23.38 µM. Analysis of the determined IC_50_ values provides us with an interesting structure–activity relationship (SAR).

The most potent inhibitors were tacrine derivatives, with the majority of compounds’ IC_50_ values in the low micromolar range (1.31–8.07 µM). Again, it highlights tacrine as a privileged structure that causes multidirectional activities of its derivatives. The activity of the compounds with the tacrine moiety was maintained in all three series, although it was most stable within 1-(phenylsulfonyl)-4-(piperazin-1-yl)-1*H*-indole derivatives (series I, Table 1). Compounds from series I all displayed µM potencies, while in series II compound **20**, with the longest linker inhibited the enzyme in 62% and in series III **28** and **30** showed 67% and 34% of inhibition, respectively. Also, among non-tacrine derivatives, the most potent BACE1 inhibitors belong to series I: **7, 13,** and **14**. Importance of 1-(phenylsulfonyl)-4-(piperazin-1-yl)-1*H*-indole fragment for interactions with BACE1 can be observed on an example of *N*-benzylamine derivatives. Comparing three analogues from each series, compound **8** (series I), **21** (series II), and **32** (series III) we can see quite similar inhibitory potency for indole-based compounds IC_50_=19.66 µM and IC_50_=21.88 µM for **8** and **21**, respectively, and decrease in activity for **32** − 42.8% at 50 µM concentration. These observations indicate an important role of 1-(phenylsulfonyl)-4-(piperazin-1-yl)-1*H*-indole, 1-benzyl-4-(piperazin-1-yl)-1*H*-indole and 1-(3-(benzyloxy)-2-methylphenyl)piperazine1-(3-(benzyloxy)-2-methylphenyl)piperazine fragments in the binding mode of the compounds within BACE1. Therefore, we chose compounds **8**, **21,** and **32** for docking studies to investigate the differences in the binding mode that could cause differences in activity.

**Figure 2.**
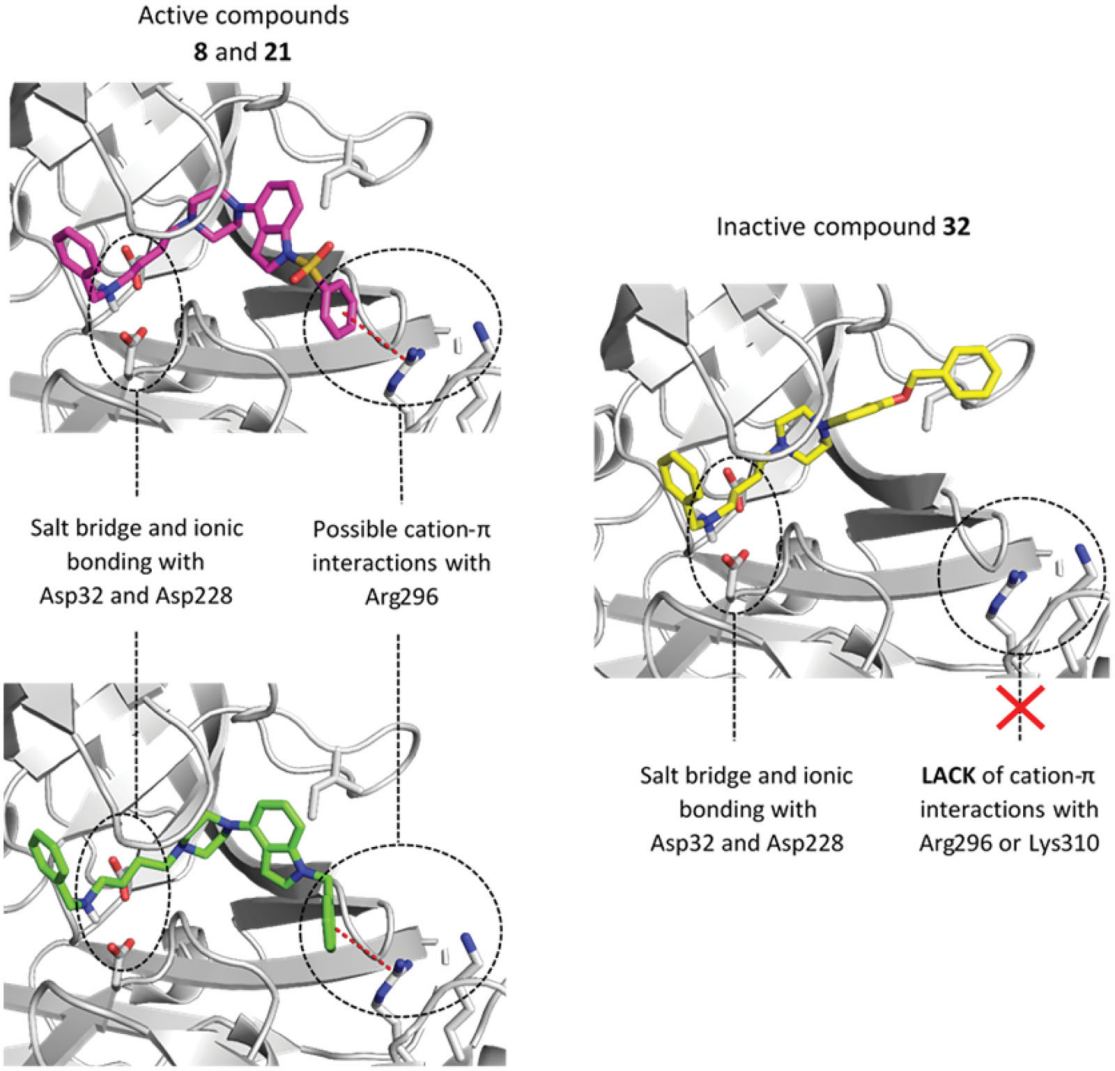
The predicted binding mode of compounds **8** (magenta), **21** (green), and **32** (yellow) in the binding site of BACE1. The detected cation-π interactions were marked as red lines connecting the centre of the aromatic ring and guanidine carbon of Arg296.

The analysis of the predicted binding mode of compounds **8**, **21,** and **32** within BACE1 active site (Figure 2), showed a uniform arrangement of the benzylamine fragment within subsites S3' and S2'. This position is consistent with X-ray crystallographic data on different BACE1 inhibitors containing the benzylamine fragment^47^^,^^48^. We found the most significant changes in the arrangement of these compounds within the S3 and S4 subsites. In the case of indole derivatives (**8**, **21**), the phenyl ring is directed towards the Arg296 and Lys310 and is engaged in a cation-π interaction. Distances between a centre of the aromatic ring and guanidine carbon of Arg296 were 5.2 Å (**8**) and 5.6 Å (**21**), respectively. In the complex used for docking, Lys 310 residue is outside the boundary for cation-π interactions (6 Å distance) (7.6 Å for comp. **8** and 7.0 Å for comp. **21**), however, due to the high conformational freedom visible in many crystal structures, Lys310 may be important for binding of the active ligands. In **32,** the benzyloxy fragment is mostly directed towards Ile110 where it creates weak, hydrophobic interactions. Additionally, the larger indole fragment creates more interactions with the S3 subsite than its bioisostere: methylphenol. Used GoldScore (GS) scoring function showed an approx. the 30-point difference in favour of indole derivatives than their reduced analogue (**8** − 62.00, **21** − 56.55, and **32** − 30.89). These results suggest that interactions within subsides S3 and S4 cause the differences in compounds activities.

**Figure 3.**
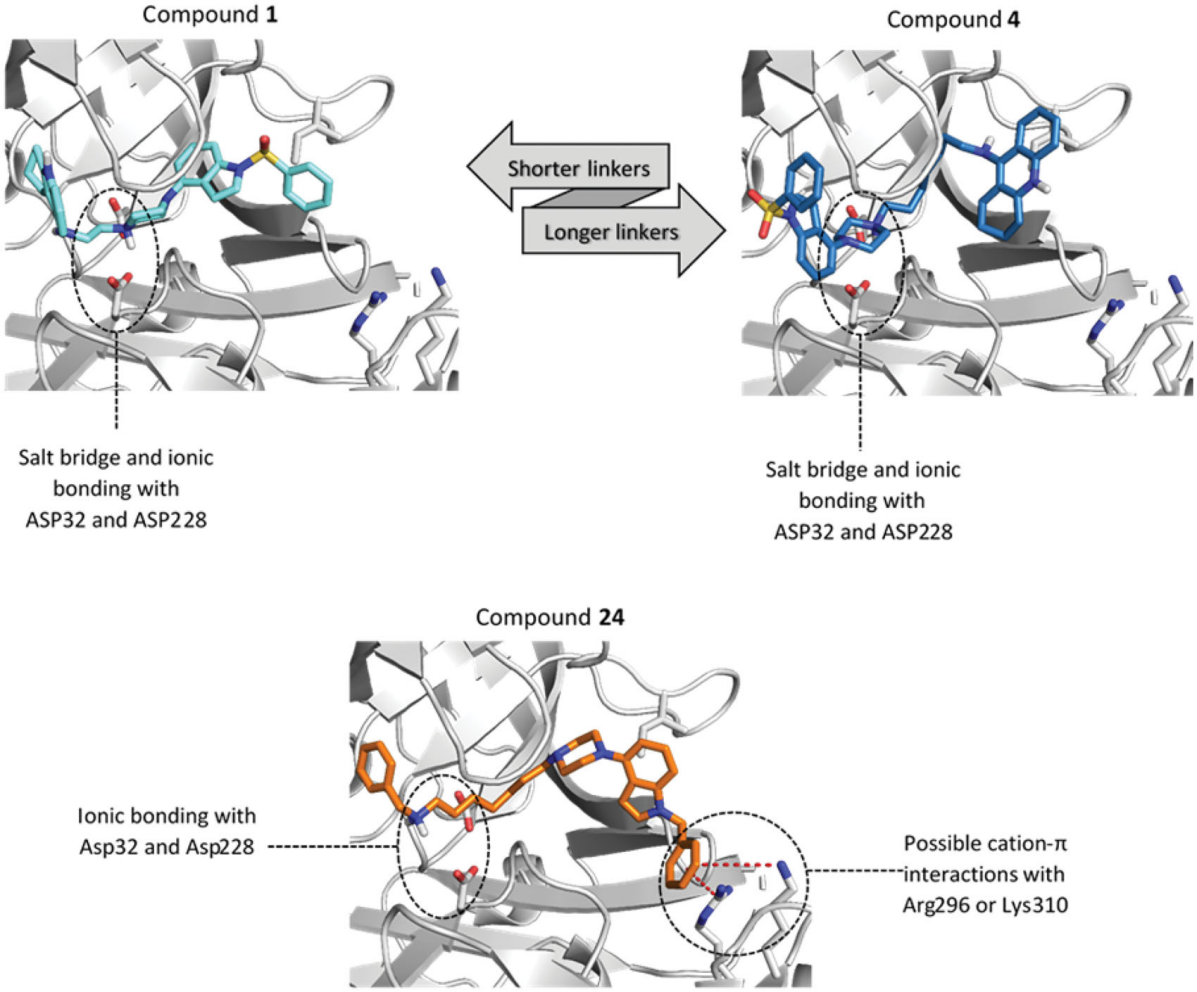
Differences in the binding mode of compounds **1** (cyan), **4** (blue) and **24** (orange) within BACE1. The detected cation-π interactions were marked as red lines connecting the centre of the aromatic ring and guanidine carbon of Arg296 (4.3 Å) or Lys310 (5.6 Å).

**Figure 4.**
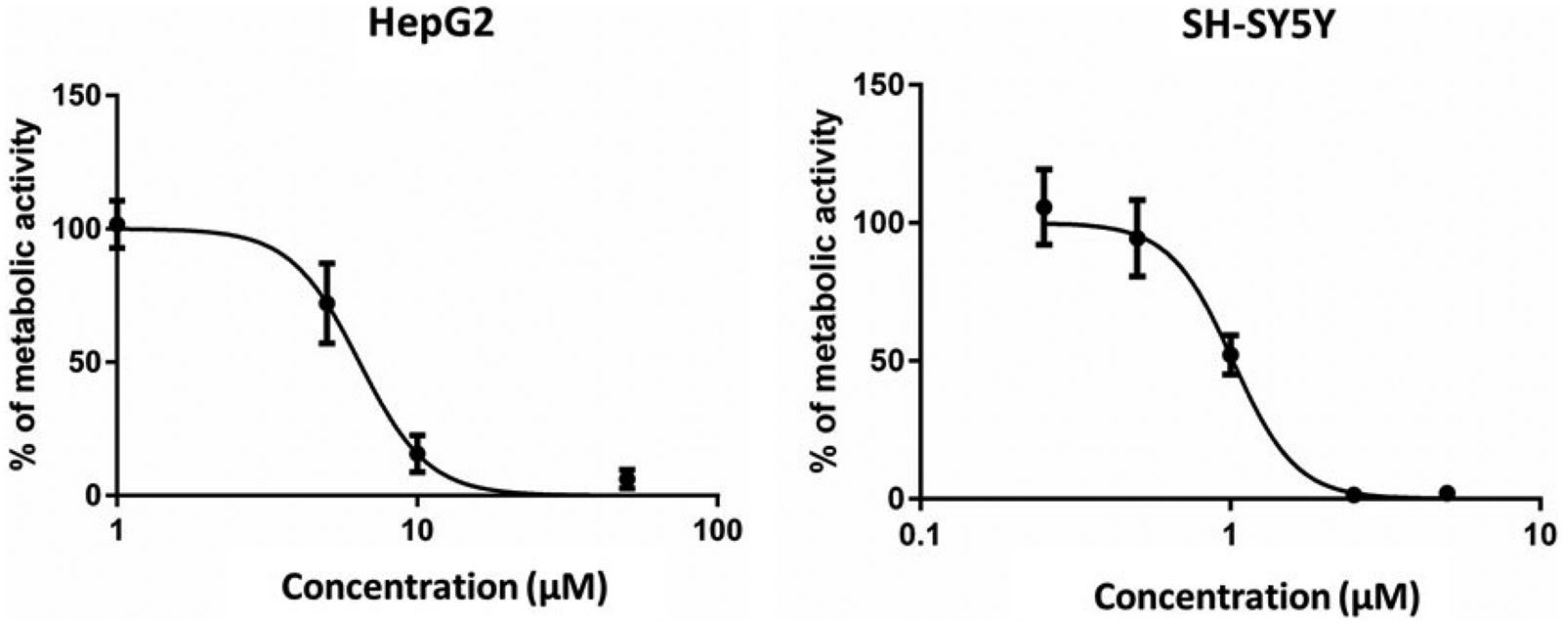
Compound **3** exerts concentration-dependent influence on the metabolic activity of HepG2 and SH-SY5Ycells. HepG2 and SH-SY5Ycells were incubated in the presence of increasing concentrations of compound **3** (1–100 µM) and (0.25–5 µM), respectively. After 24 h, cell viability was evaluated by MTS assay. A control group (DMSO) was considered as 100% of cell viability. Cells were treated in quadruplicate. The values are the mean ± SD of two independent experiments.

The influence of the linker length on the binding mode and the activity the tested compounds depends largely on the 5-HT_6_ and ChE-targeting fragments connected. We have observed two distinct binding modes analysing the docking results of the two most active tacrine derivatives with two (compound **1**) and seven carbon atom linkers (compound **4**). In both, the key factor is the formation of a salt bridge between the protonated nitrogen atom of the piperazine and the catalytic dyad (Figure 3). In the case of compounds with a short linker like compound **1**, we see tacrine in pockets S2' and S3' and the indole system interacting with the amino acids building the pockets S1–S3 (Figure 4). This position was maintained in all analogous structures with a linker length of up to five carbon atoms. Compounds with the longer linkers (6–8 carbon atoms) show the opposite arrangement as shown on the example of compound **4** (Figure 3). The indole system is now placed within pockets S2' and S3' and the linker remains rolled up in the hydrophobic space of the pocket S1 directing tacrine to the pocket S2 and S3. This change only slightly affects the formation of a salt bridge between the piperazine system and the catalytic dyad. Both arrangements are similar in terms of the number of specific bonds, such as hydrogen bonds, polar, or aromatic interactions. We have not observed such difference in the binding mode of benzylamine derivatives, which may result from the predominant role of interactions with Asp32 and Asp228.

### 2.4. Structure–activity relationship analysis

We have described in details SAR of all the compounds in terms of their activity against 5-HT_6_ receptor and cholinesterases previously^30^^,^^31^. Briefly, SAR analysis led to the identification of a 1-(phenylsulfonyl)-4-(piperazin-1-yl)-1*H*-indole as a fragment that provides stable and strong affinity for 5-HT_6_ receptor regardless the cholinesterase-targeting fragment or the length of the linker (*K*_i_=2–39 nM). The other 5-HT_6_-targeting moieties: 1-benzyl-4-(piperazin-1-yl)-1*H*-indole and 1-(3-(benzyloxy)-2-methylphenyl)piperazine1-(3-(benzyloxy)-2-methylphenyl)piperazine turned out to be much more sensitive to different substituents on the piperazine ring leading to decreased affinities for the receptor (*K*_i_=72–916 nM for series II; *K*_i_=18–845 nM for series III), with the highest affinities found for tacrine derivatives. In all three series of compounds tacrine moiety provided high, nanomolar potency towards both AChE and BuChE regardless of the linker (AChE IC_50_=5–57 nM and BuCHE IC_50_=5–45 nM). Herein, we have shown that tacrine, especially in combination with 1-(phenylsulfonyl)-4-(piperazin-1-yl)-1*H*-indole, contribute also to potent inhibition of BACE1 (IC_50_=1–8 µM). The potency against cholinestereases and BACE1 is more varied for *N*-benzylamine, *N*-benzylpiperidine, and *N*-benzylpyrrolidine derivatives. In terms of BACE1 inhibition some of these compounds (**7**, **13**, or **14**) show activity like those of tacrine derivatives and the others like compounds **33**, **36**, **38** inhibit the enzyme in only about 30% at 50 µM concentration. It also indicates the positive influence of 1-(phenylsulfonyl)-4-(piperazin-1-yl)-1*H*-indole fragment present in compounds **7**, **13,** and **14**. The influence of the linker on the compounds’ activity towards BACE1 cannot be indicated, it depends on the combination of the fragments in each compound.

### 2.5. Cytotoxicity studies

We have determined the hepatotoxic and neurotoxic effects of compound **3** which is a representative of the most potent series and at the same time tacrine derivative, which potentially may carry a risk of hepatotoxicity. We have treated liver hepatocellular HepG2 and neuroblastoma SH-SY5Y cells with compound **3** for 24 h and determined cell viability assessed by MTS assay. We have observed a concentration-dependent decrease of cells’ viability with LC_50_ values of 6.4 and 1.0 µM on HepG2 and SH-SY5Y cells, respectively (Figure 4). The LC_50_ values were significantly higher than the doses effective against 5-HT_6_ or cholinesterases (5-HT_6_ *K*_i_ =2 nM, *h*AChE IC_50_=13 nM and *eq*BuChE IC_50_=8 nM), but in the similar ranges to those active against BACE1 (IC_50_=7.3 µM), tau (38% at 10 µM), and amyloid β (30% at 10 µM). Therefore, in the course of the development of these compounds’ their toxicity will require further investigation.

## 3. Conclusions

AD is the main cause of dementia, sixth leading cause of death and from a perspective of pharmacotherapy unsolved problem and a huge challenge. Disorders in neurotransmission, especially in the cholinergic system, observed in patients with AD, led to the development of cholinesterase inhibitors as anti-AD drugs. In addition to the symptomatic intervention resulting from inhibition of cholinesterases, it was shown that they might display a disease-modifying effect due to the modulation of Aβ aggregation. Aggregation of Aβ and tau protein can be detected far ahead of the onset of disease symptoms. Therefore, inhibition or modulation of these processes is considered a major approach towards disease-modifying therapeutics.

Recently, we have developed *in vitro* and *in vivo* potent multifunctional ligands with BuChE inhibitory activity and 5-HT_6_ antagonism. Due to the interesting activity profile of these compounds, we decided to carry on an extended *in vitro* and *in cellulo* tests to verify their disease-modifying potential. We have tested them against crucial biological targets that contribute to the development of the disease: Aβ and tau protein aggregation and BACE1. These studies led to the emergence of a group of compounds that affect not only neurotransmission but also inhibit processes of Aβ and tau aggregation. SAR studies led to the conclusion that 1-(phenylsulfonyl)-4-(piperazin-1-yl)-1*H*-indole and tacrine are privileged structures that provide a broad profile of biological activities, including inhibitory potency against tau and Aβ aggregation in *in cellulo* assay ranging from 30% to 61% at 10 µM, *h*BACE1 with IC_50_ values from 2 to 8 µM, *h*5-HT_6_ *K*_i_ values between 2 and 36 nM, *h*AChE IC_50_ values between 1 and 46 nM and *eq*BuChE IC_50_ values in ranges from 8 to 21 nM. Also, noteworthy are non-tacrine derivatives **16** and **37**–**39** due to their potent anti-aggregating properties *in cellulo*, 57–63% against tau and 54–62% against Aβ. Compounds that inhibit both amyloid β and tau aggregation are in the minority among the multifunctional ligands against AD. Therefore, multifunctional ligands reported herein, not only potently inhibiting proteins’ aggregation but also displaying BACE1 and BuChE inhibitory potency and 5-HT_6_ antagonistic properties can be of great value for the development of new therapeutic strategies.

The study led to the emergence of multifunctional ligands with disease-modifying and symptomatic effects in *in vitro* and *in cellulo* models that are an excellent starting point for further search for anti-Alzheimer’s therapy.

## 4. Methods

### 4.1. Inhibition of Aβ_42_/tau aggregation in *Escherichia coli* overexpressing Aβ_42_/tau

#### 4.1.1 Chemicals and bacterial culture media

The assays were conducted according to the procedure described previously^35^. All general chemicals were purchased from Sigma-Aldrich (St. Louis, MO) . Reagents for bacterial media were purchased from Conda (Sevilla, Spain). M9 minimal medium for 100 mL: 10 mL of salts 10× (0.68 g of Na_2_HPO_4_, 0.30 g of KH_2_PO_4_, 0.05 g of NaCl, 0.10 g of NH_4_Cl), 0.2 mL of 1 M MgSO_4_, 0.2 mL of 50 mM CaCl_2_, 2.5 mL of 20% glucose, and 87.1 mL of H_2_O.

#### 4.1.2. Aβ42/tau aggregation inhibition assay in *Escherichia coli*

*E. coli* competent cells BL21 (DE3) were transformed with the pET28a vector (Novagen, Inc., Madison, WI) carrying the DNA sequence of Aβ_42_ or with pTARA containing the RNA polymerase gene of T7 phage (T7RP) under the control of the promoter pBAD, then with pRKT42 vector encoding four repeats of tau protein in two inserts. For overnight culture preparation, 10 ml of M9 minimal medium containing 50 μg/mL of kanamycin (Aβ_42_) or 50 μg/mL of ampicillin and 12.5 μg/mL of chloramphenicol (tau) were inoculated with a colony of BL21 (DE3) bearing the plasmid Aβ_42_ or tau plasmids to be expressed at 37 °C and 180 rpm using an incubator shaker (Ovan, Barcelona, Spain). After overnight growth, the OD_600_ was usually 2 − 2.5. For expression of Aβ_42_ peptide/tau protein, 200 μL of overnight culture were transferred into Eppendorf tubes of 1.5 mL containing 790 μL of fresh M9 minimal medium with 50 μg/mL of kanamycin1 mM of isopropyl 1-thio-β-D-galactopyranoside (IPTG) (Aβ_42_) or 50 μg/mL of ampicillin, 12.5 μg/mL of chloramphenicol, 0.25% of arabinose (tau), and 25 μM of ThS and 10 μL of reference, tested compounds (1 mM in DMSO, final concentration: 10 μM) or DMSO. The samples were grown for 24 h at 37 °C and 180 rpm using an incubator shaker (Ovan). The samples with DMSO were prepared as a negative control (maximum level of Aβ_42_/tau, 0% of inhibition). In the positive controls, the M9 medium without IPTG (minimal level of Aβ_42_) or arabinose (minimal level of tau) was used. For absorbance and fluorescence determination, 200 μL of each Eppendorf tube was transferred in triplicate into 96-well plate. Finally, the fluorescence emission at 520 nm, when exciting at 440 nm and absorbance at 600 nm was recorded using the multi-mode microplate reader (Beckman Counter, Brea, CA). The absorbance at 600 nm (OD_600_) of these samples was assessed to check the bacterial growth and potential intrinsic toxicity of the tested compounds. Final data are the average of three independent experiments. **DP-128** was used as a reference compound^38^.

### 4.2. *In vitro* inhibition of human BACE1

BACE1 FRET Assay Kit (Panvera, Madison, WI) was performed according to the manufacturer’s protocol with small modifications. The Kit includes purified baculovirus-expressed BACE1 and specific peptide substrate (Rh-EVNLDAEFK-quencher), based on the “Swedish” mutant of APP. The analysis was carried, using 384-well black microplates and a microplate reader (EnSpire Multimode; PerkinElmer, Waltham, MA). The wavelength was optimised for the 553 nm excitation and 576 nm emission. Stock solutions of all test compounds were prepared in DMSO and further diluted with assay buffer (50 mM sodium acetate; pH 4.5). In the first step, 10 μL of BACE1 substrate was mixed with 10 μL of test compound (or assay buffer; i.e. blank sample), then 10 μL of the enzyme (1 U/mL) was added to start the reaction. After 60 min of incubation at 25 °C, 10 μL of stop solution (2.5 M sodium acetate) was applied to stop the reaction. The fluorescence signal was read at 576 nm. Percent of inhibition was calculated from [1 − (S60 − S0)/(C60 − C0)] × 100, where S0 and S60 are fluorescence intensities of the test sample (enzyme, substrate, test compound) at the beginning of the reaction and after 60 min, respectively, while C0 and C60 are analogical fluorescence intensities of the blank sample (enzyme, substrate, buffer). All the compounds were tested at a screening concentration of 50 μM. Each compound was analysed in triplicate. Commercially available BACE1 Inhibitor IV (Calbiochem, Merck; Nottingham, UK) was used as the reference compound. The IC_50_ values were calculated using nonlinear regression (GraphPad Prism 5 [GraphPad Software, San Diego, CA, 5.00]) by plotting the residual enzyme activities against the applied inhibitor concentration.

### 4.3. Cytotoxicity studies for compound 3

#### 4.3.1. Cell culture and treatments

The human liver carcinoma HepG2 cell line was purchased from American Type Culture Collection (LGC Standards, UK). The human neuroblastoma SH-SY5Y cell line was purchased from American Type Culture Collection (CRL-2266, Manassas, VA). The cells were grown in Advanced Dulbecco’s modified Eagle’s medium (Sigma, St. Louis, MO) supplemented with 10% foetal bovine serum (FBS) (HyClone, Logan, UT), 2 mM *L*-glutamine, 50 U/mL penicillin and 50 μg/mL streptomycin (Sigma, St. Louis, MO) in a humidified atmosphere of 95% air and 5% CO_2_ at 37 °C, and grown to 80% confluence. Compound 3 was prepared as a stock solution of 20 mM in DMSO and was used at concentrations of 1–100 µM in case of HepG2 treatment, and at concentrations of 0.25–5 µM in case of SH-SY5Y treatment.

#### 4.3.2. Cell viability assay

HepG2 and SH-SY5Y cells were seeded in 96-well plates (2 × 10^4^/well) and assessed by MTS ([3–(4,5-dimethylthiazol-2-yl)-5–(3-carboxymethoxyphenyl)-2–(4-sulfophenyl)-2H-tetrazolium, inner salt) assay for their response to compound 3. Cells were treated as described above, and cell viability was assessed after 24 h using the CellTiter 96^®^ Aqueous One Solution Cell Proliferation Assay (Promega, Madison, WI), following the manufacturer’s instructions. Absorbance was measured with an automatic microplate reader (Tecan Safire, Switzerland) at a wavelength of 492 nm. Results are presented as a percentage of the control (DMSO).

### 4.4. Molecular modelling studies

All tested compounds were prepared in LigPrep (Schrödinger) and exported to mol2 files. Docking studies were performed with GOLD 5.3 (CCDC). Before docking, BACE1 protein (PDB code 4D8C) was prepared using Hermes 1.7 (CCDC). All histidine residues were protonated at Nε, the hydrogen atoms were added, a ligand and water molecules were removed. The binding sites were defined as all amino acid residues within a radius of 15 Å from ligand (BXD) in the BACE1 complex. We applied the automatic settings of the genetic algorithm for very flexible ligands (number of operations equal 125,000). We received 10 conformations by the ligand sorted by the GoldScore scoring function. Results of docking were visualised with PyMOL 0.99rc6 (DeLano Scientific LLC, San Francisco, CA).
